# Crystal structure of 7-[(2*E*)-2-benzyl­idene-3-oxobut­oxy]-4-methyl-2*H*-chromen-2-one

**DOI:** 10.1107/S2056989015003084

**Published:** 2015-03-04

**Authors:** Ignez Caracelli, Julio Zukerman-Schpector, Paulo J. S. Moran, Bruno R. S. de Paula, Edward R. T. Tiekink

**Affiliations:** a Departmento de Física, Universidade Federal de São Carlos, 13565-905 São Carlos, SP , Brazil; b Departmento de Química, Universidade Federal de São Carlos, 13565-905 São Carlos, SP, Brazil; c Instituto de Química, Universidade Estadual de Campinas, C.P. 6154, 13083-970 Campinas, SP, Brazil; d Department of Chemistry, University of Malaya, 50603 Kuala Lumpur, Malaysia

**Keywords:** crystal structure, chromen-2-one, conformation

## Abstract

Two independent mol­ecules (*A* and *B*) comprise the asymmetric unit of the title compound, C_21_H_18_O_4_. There are significant conformational differences between the mol­ecules relating in particular to the relative orientation of the 3-oxo-2-(phenyl­methyl­idene)but­oxy substituent with respect to the superimposable chromen-2-one residues. To a first approximation, the substituents are mirror images; both are approximately perpendicular to the chromen-2-one fused ring system with dihedral angles of 88.50 (7) (*A*) and 81.96 (7)° (*B*). Another difference between the independent mol­ecules is noted in the dihedral angles between the adjacent phenyl and but-3-en-2-one groups of 8.72 (12) (*A*) and 27.70 (10)° (*B*). The conformation about the ethene bond in both mol­ecules is *E*. The crystal packing features C—H⋯O, C—H⋯π(ar­yl) and π–π [*Cg*⋯*Cg* = 3.6657 (8) and 3.7778 (8) Å] stacking inter­actions, which generate a three-dimensional network.

## Related literature   

For background to the biotransformation procedure mediated by *Saccharomyces cerevisiae*, see: de Paula *et al.* (2013 ▸). For the structure of the closely related compounds 7-all­yloxy-2*H*-chromen-2-one and (3*E*)-3-(4-nitro­phen­oxy­meth­yl)-4-phenyl­but-3-en-2-one, see: Seth *et al.* (2011 ▸); Zukerman-Schpector *et al.* (2014 ▸).
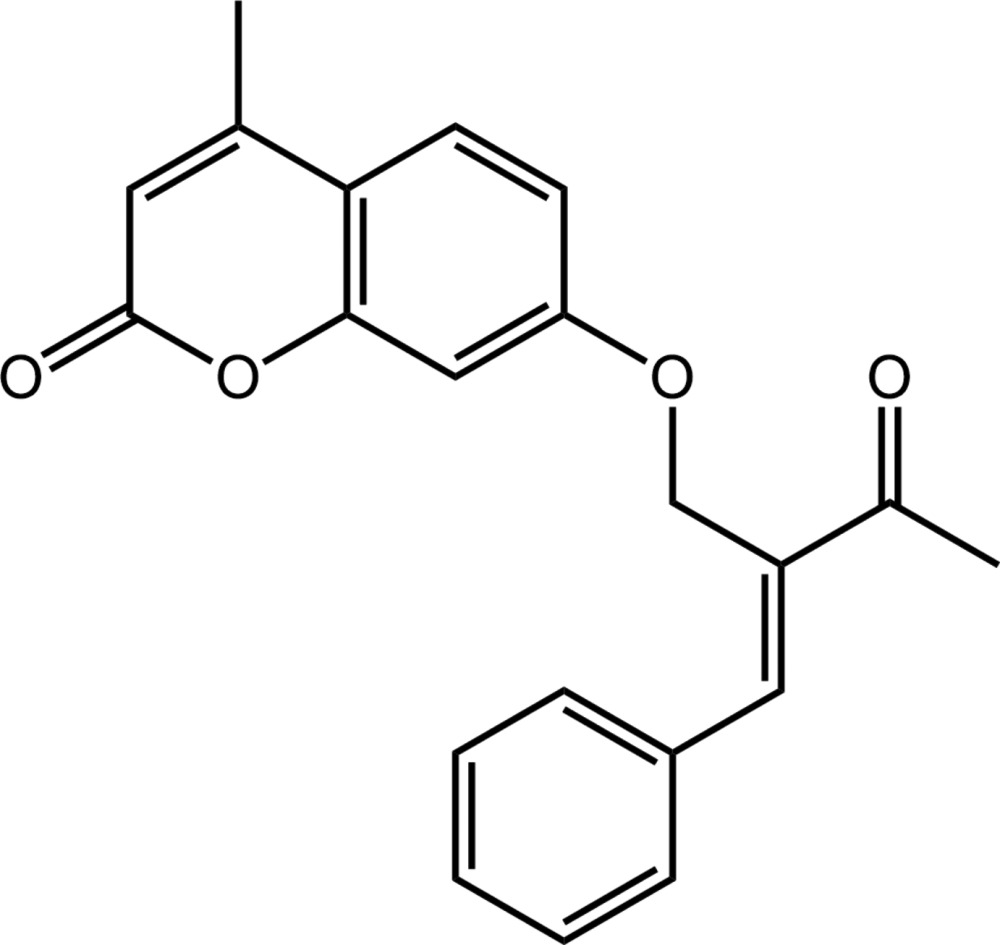


## Experimental   

### Crystal data  

C_21_H_18_O_4_
*M* *_r_* = 334.36
Triclinic, 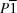

*a* = 9.7755 (8) Å
*b* = 12.3986 (10) Å
*c* = 14.1827 (11) Å
α = 86.293 (3)°
β = 84.328 (2)°
γ = 86.816 (2)°
*V* = 1704.9 (2) Å^3^
*Z* = 4
Mo *K*α radiation
μ = 0.09 mm^−1^
*T* = 296 K
0.52 × 0.38 × 0.33 mm

### Data collection  

Bruker APEXII CCD diffractometer
Absorption correction: multi-scan (*SADABS*; Sheldrick, 1996 ▸) *T*_min_ = 0.664, *T*_max_ = 0.745
18494 measured reflections
6263 independent reflections
5296 reflections with *I* > 2σ(*I*)
*R*_int_ = 0.022

### Refinement  

*R*[*F* ^2^ > 2σ(*F* ^2^)] = 0.042
*wR*(*F* ^2^) = 0.124
*S* = 1.05
6263 reflections
456 parameters
H-atom parameters constrained
Δρ_max_ = 0.34 e Å^−3^
Δρ_min_ = −0.20 e Å^−3^

### 

Data collection: *APEX2* (Bruker, 2009 ▸); cell refinement: *SAINT* (Bruker, 2009 ▸); data reduction: *SAINT*; program(s) used to solve structure: *SIR2014* (Burla *et al.*, 2015 ▸); program(s) used to refine structure: *SHELXL2014* (Sheldrick, 2015 ▸); molecular graphics: *ORTEP-3 for Windows* (Farrugia, 2012 ▸), *QMOL* (Gans & Shalloway, 2001 ▸) and *DIAMOND* (Brandenburg, 2006 ▸); software used to prepare material for publication: *MarvinSketch* (ChemAxon, 2010 ▸) and *publCIF* (Westrip, 2010 ▸).

## Supplementary Material

Crystal structure: contains datablock(s) I, New_Global_Publ_Block. DOI: 10.1107/S2056989015003084/hb7368sup1.cif

Structure factors: contains datablock(s) I. DOI: 10.1107/S2056989015003084/hb7368Isup2.hkl

Supporting information file. DOI: 10.1107/S2056989015003084/hb7368Isup3.cml

**Fig. 1.**
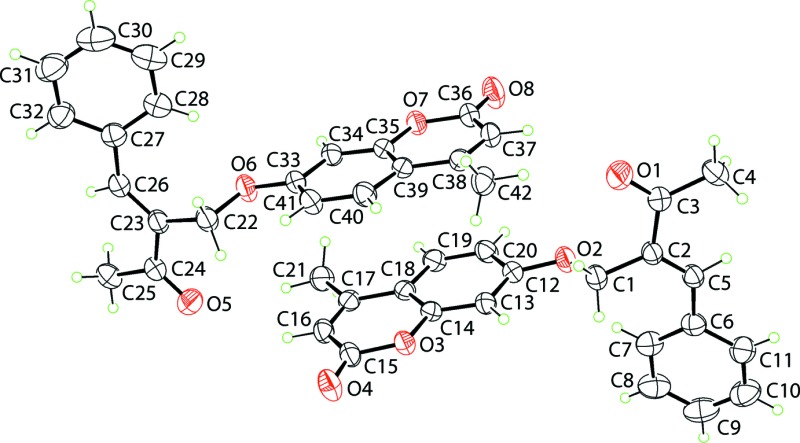
The molecular structures of the two independent molecules in title compound showing the atom-labelling scheme and displacement ellipsoids at the 35% probability level.

. DOI: 10.1107/S2056989015003084/hb7368fig1.tif
The mol­ecular structures of the two independent mol­ecules in title compound showing the atom-labelling scheme and displacement ellipsoids at the 35% probability level.

**Fig. 2.**
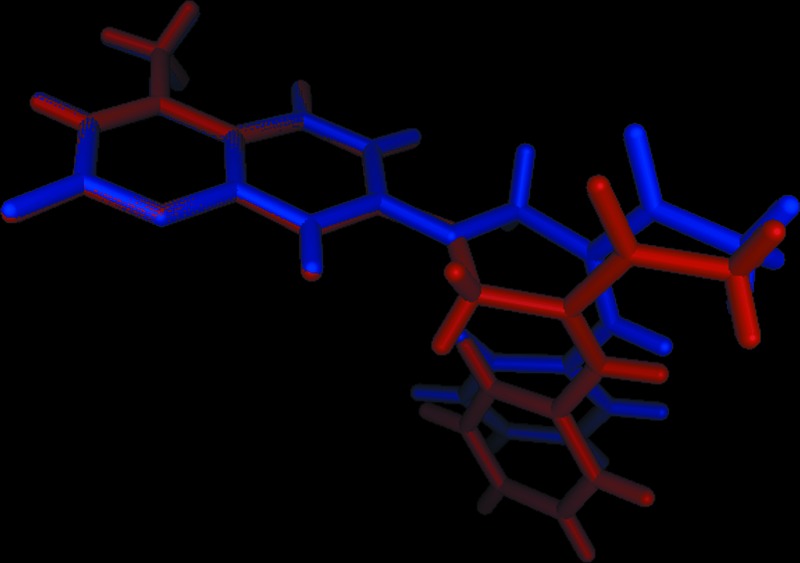
Superimposition of the two independent molecules. Molecule A is shown in red and inverted B in blue. The molecules have been superimposed such that the chromen-2-one rings are overlapped.

A B . DOI: 10.1107/S2056989015003084/hb7368fig2.tif
Superimposition of the two independent mol­ecules. Mol­ecule *A* is shown in red and inverted *B* in blue. The mol­ecules have been superimposed such that the chromen-2-one rings are overlapped.

**Fig. 3.**
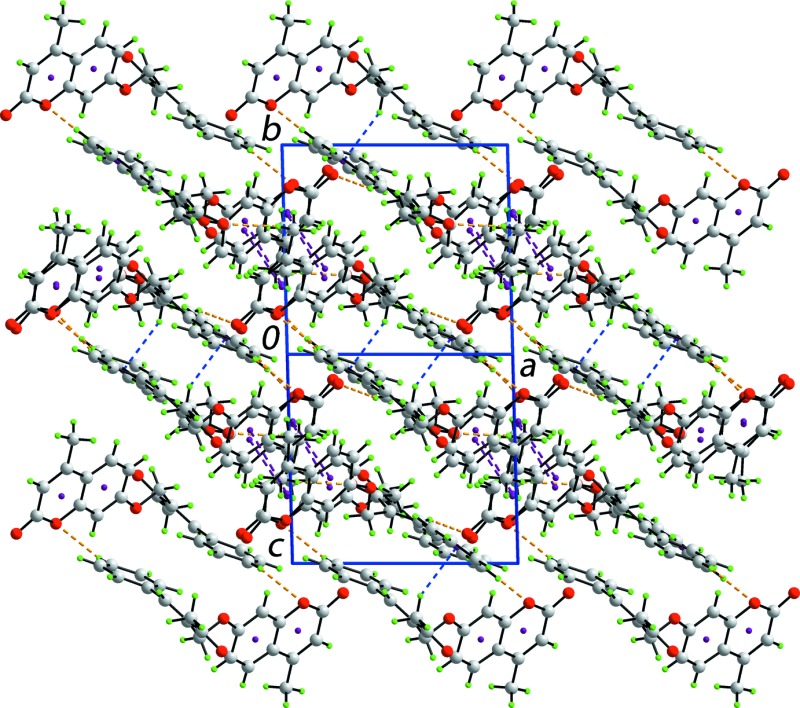
A view in projection down [0 1 1] of the unit-cell contents. The C—H···O, C—H···π and π—π interactions are shown as orange, blue and purple dashed lines, respectively.

. DOI: 10.1107/S2056989015003084/hb7368fig3.tif
A view in projection down [0 1 1] of the unit-cell contents. The C—H⋯O, C—H⋯π and π—π inter­actions are shown as orange, blue and purple dashed lines, respectively.

CCDC reference: 1049265

Additional supporting information:  crystallographic information; 3D view; checkCIF report

## Figures and Tables

**Table 1:** Hydrogen-bond geometry (, ) *Cg*1 is centroid of the C6C11 ring.

*D*H*A*	*D*H	H*A*	*D* *A*	*D*H*A*
C5H5O8^i^	0.93	2.59	3.4778(19)	159
C9H9O3^ii^	0.93	2.52	3.381(2)	155
C30H30O7^iii^	0.93	2.59	3.441(2)	153
C42H42*B*O1	0.96	2.53	3.427(2)	155
C25H25*C* *Cg*1^iv^	0.96	2.64	3.5238(18)	152

Symmetry codes: (i) 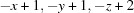; (ii) 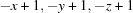; (iii) 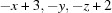; (iv) 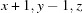.

## supplementary crystallographic information

### S3. Comment

These title compound was synthetized to be used as a substrate in the modelling of the biotransformation procedure mediated by *Saccharomyces cerevisiae*,specifically in order to compare the role of different leaving groups (de Paula *et al.*, 2013). The goal was to develop a procedure that could follow the reaction progress by colorimetric or fluorescence techniques.

### S4. Experimental

4-Methylumbelliferone (352 mg, 2 mmol) and potassium carbonate (232 mg, 2.4 mmol) were added to a solution of (*E*)-3-bromomethyl-4-phenyl-3-buten-2-one (478 mg, 2 mmol) in dry acetone (4 ml). The solution was stirred for 3 h, and then filtered. The solvent was evaporated, and the residue was recrystallized from a CH_2_Cl_2_/hexane mixture to afford the product as a colourless solid in 63% yield. The product was recrystallized by slow evaporation of a 3:1 CH_2_Cl_2_/hexane mixture. *M*.pt 188.1–189.1 °C. ^1^H NMR (CD_2_Cl_2_, 400 MHz): δ 2.40 (d, 3H, J = 1.2 Hz), 2.51 (s, 3H), 4.88 (s, 2H), 6.10 (q, 1H, J = 1.2 Hz), 6.87 (d, 1H, J = 2.4 Hz), 6.92 (dd, 1H, J = 2.4, 8.8 Hz), 7.38–7.51 (m, 5H), 7.55 (d, 1H, J = 8.8 Hz), 7.91 (s, 1H). ^13^C NMR (CD_2_Cl_2_, 150 MHz): δ 19.0, 26.4, 62.7, 102.5, 112.6, 113.0, 114.6, 126.3, 129.4, 130.2, 130.4, 135.0, 135.8, 146.3, 153.2, 155.8, 161.4, 162.0, 198.6.

### S5. Refinement

Carbon-bound H-atoms were placed in calculated positions (C—H = 0.93–0.97 Å) and were included in the refinement in the riding model approximation, with *U*_iso_(H) = 1.2 *U*_eq_(C).

### Crystal data

**Table:**

C_21_H_18_O_4_	*Z* = 4
*M**_r_* = 334.36	*F*(000) = 704
Triclinic, *P*1	*D*_x_ = 1.303 Mg m^−^^3^
*a* = 9.7755 (8) Å	Mo *K*α radiation, λ = 0.71073 Å
*b* = 12.3986 (10) Å	Cell parameters from 9897 reflections
*c* = 14.1827 (11) Å	θ = 2.7–25.4°
α = 86.293 (3)°	µ = 0.09 mm^−^^1^
β = 84.328 (2)°	*T* = 296 K
γ = 86.816 (2)°	Slab, colourless
*V* = 1704.9 (2) Å^3^	0.52 × 0.38 × 0.33 mm

### Data collection

**Table:**

Bruker APEXII CCD diffractometer	5296 reflections with *I* > 2σ(*I*)
φ and ω scans	*R*_int_ = 0.022
Absorption correction: multi-scan (*SADABS*; Sheldrick, 1996)	θ_max_ = 25.5°, θ_min_ = 1.5°
*T*_min_ = 0.664, *T*_max_ = 0.745	*h* = −11→11
18494 measured reflections	*k* = −14→11
6263 independent reflections	*l* = −17→12

### Refinement

**Table:**

Refinement on *F*^2^	Hydrogen site location: inferred from neighbouring sites
Least-squares matrix: full	H-atom parameters constrained
*R*[*F*^2^ > 2σ(*F*^2^)] = 0.042	*w* = 1/[σ^2^(*F*_o_^2^) + (0.0611*P*)^2^ + 0.3298*P*] where *P* = (*F*_o_^2^ + 2*F*_c_^2^)/3
*wR*(*F*^2^) = 0.124	(Δ/σ)_max_ = 0.001
*S* = 1.05	Δρ_max_ = 0.34 e Å^−^^3^
6263 reflections	Δρ_min_ = −0.20 e Å^−^^3^
456 parameters	Extinction correction: *SHELXL2014* (Sheldrick, 2015), Fc^*^=kFc[1+0.001xFc^2^λ^3^/sin(2θ)]^-1/4^
0 restraints	Extinction coefficient: 0.0232 (15)

### Special details

**Table:**

Geometry. All e.s.d.'s (except the e.s.d. in the dihedral angle between two l.s. planes) are estimated using the full covariance matrix. The cell e.s.d.'s are taken into account individually in the estimation of e.s.d.'s in distances, angles and torsion angles; correlations between e.s.d.'s in cell parameters are only used when they are defined by crystal symmetry. An approximate (isotropic) treatment of cell e.s.d.'s is used for estimating e.s.d.'s involving l.s. planes.

### Fractional atomic coordinates and isotropic or equivalent isotropic displacement parameters (Å^2^)

**Table:**

	*x*	*y*	*z*	*U*_iso_*/*U*_eq_	
C1	0.66167 (15)	0.54439 (11)	0.77694 (10)	0.0509 (3)	
H1A	0.6424	0.5688	0.7130	0.061*	
H1B	0.7553	0.5612	0.7853	0.061*	
C2	0.56313 (14)	0.60008 (11)	0.84772 (9)	0.0461 (3)	
C3	0.61877 (16)	0.61816 (12)	0.93888 (10)	0.0552 (4)	
C4	0.53887 (19)	0.68481 (15)	1.01131 (11)	0.0697 (5)	
H4A	0.5969	0.6995	1.0593	0.105*	
H4B	0.4616	0.6460	1.0400	0.105*	
H4C	0.5067	0.7518	0.9813	0.105*	
C5	0.43370 (14)	0.63270 (11)	0.83276 (10)	0.0482 (3)	
H5	0.3868	0.6720	0.8806	0.058*	
C6	0.35438 (15)	0.61632 (12)	0.75311 (10)	0.0525 (3)	
C7	0.37769 (18)	0.52938 (13)	0.69523 (11)	0.0614 (4)	
H7	0.4502	0.4793	0.7047	0.074*	
C8	0.2945 (2)	0.51655 (16)	0.62389 (13)	0.0760 (5)	
H8	0.3118	0.4582	0.5857	0.091*	
C9	0.1871 (2)	0.58891 (19)	0.60901 (14)	0.0851 (6)	
H9	0.1307	0.5793	0.5614	0.102*	
C10	0.1628 (2)	0.67582 (19)	0.66457 (15)	0.0836 (6)	
H10	0.0908	0.7259	0.6536	0.100*	
C11	0.24423 (17)	0.68949 (16)	0.73662 (13)	0.0680 (4)	
H11	0.2256	0.7480	0.7746	0.082*	
C12	0.73601 (14)	0.36282 (11)	0.74086 (9)	0.0462 (3)	
C13	0.83798 (14)	0.39762 (11)	0.67315 (9)	0.0450 (3)	
H13	0.8479	0.4709	0.6573	0.054*	
C14	0.92491 (13)	0.31981 (11)	0.62973 (9)	0.0415 (3)	
C15	1.11843 (15)	0.29105 (12)	0.51475 (10)	0.0491 (3)	
C16	1.10802 (15)	0.17735 (12)	0.53629 (10)	0.0507 (3)	
H16	1.1712	0.1300	0.5048	0.061*	
C17	1.01082 (15)	0.13580 (11)	0.60016 (9)	0.0469 (3)	
C18	0.91298 (14)	0.20950 (11)	0.64932 (9)	0.0430 (3)	
C19	0.80687 (15)	0.17767 (11)	0.71703 (10)	0.0506 (3)	
H19	0.7950	0.1044	0.7317	0.061*	
C20	0.72030 (16)	0.25230 (12)	0.76206 (10)	0.0526 (4)	
H20	0.6505	0.2295	0.8071	0.063*	
C21	1.00109 (18)	0.01671 (12)	0.62083 (11)	0.0619 (4)	
H21A	1.0740	−0.0209	0.5839	0.093*	
H21B	0.9139	−0.0050	0.6048	0.093*	
H21C	1.0090	−0.0005	0.6871	0.093*	
C22	1.42046 (16)	0.01415 (12)	0.66110 (11)	0.0550 (4)	
H22A	1.4946	0.0503	0.6846	0.066*	
H22B	1.3872	0.0585	0.6084	0.066*	
C23	1.47095 (15)	−0.09526 (11)	0.62931 (10)	0.0494 (3)	
C24	1.40983 (15)	−0.12992 (12)	0.54516 (10)	0.0534 (4)	
C25	1.45639 (18)	−0.23594 (13)	0.50425 (11)	0.0636 (4)	
H25A	1.5537	−0.2367	0.4862	0.095*	
H25B	1.4088	−0.2450	0.4494	0.095*	
H25C	1.4364	−0.2939	0.5509	0.095*	
C26	1.56948 (14)	−0.15546 (11)	0.66955 (10)	0.0485 (3)	
H26	1.5945	−0.2206	0.6420	0.058*	
C27	1.64397 (16)	−0.13415 (12)	0.75052 (10)	0.0531 (4)	
C28	1.59257 (19)	−0.06977 (15)	0.82394 (11)	0.0681 (4)	
H28	1.5032	−0.0395	0.8250	0.082*	
C29	1.6727 (2)	−0.05022 (17)	0.89526 (13)	0.0841 (6)	
H29	1.6376	−0.0057	0.9431	0.101*	
C30	1.8031 (2)	−0.0959 (2)	0.89606 (15)	0.0893 (6)	
H30	1.8573	−0.0813	0.9436	0.107*	
C31	1.8537 (2)	−0.16322 (19)	0.82661 (15)	0.0864 (6)	
H31	1.9413	−0.1961	0.8282	0.104*	
C32	1.77542 (17)	−0.18246 (15)	0.75440 (13)	0.0671 (4)	
H32	1.8109	−0.2283	0.7076	0.081*	
C33	1.24785 (15)	0.08942 (11)	0.77207 (9)	0.0476 (3)	
C34	1.13865 (15)	0.07032 (11)	0.83951 (9)	0.0479 (3)	
H34	1.1158	−0.0001	0.8588	0.057*	
C35	1.06426 (14)	0.15711 (11)	0.87758 (9)	0.0433 (3)	
C36	0.87152 (16)	0.21257 (12)	0.98414 (10)	0.0538 (4)	
C37	0.90271 (15)	0.32257 (12)	0.95757 (10)	0.0526 (4)	
H37	0.8461	0.3775	0.9843	0.063*	
C38	1.00978 (14)	0.34977 (11)	0.89585 (9)	0.0464 (3)	
C39	1.09595 (14)	0.26388 (11)	0.85298 (9)	0.0435 (3)	
C40	1.20862 (15)	0.27995 (12)	0.78721 (10)	0.0515 (3)	
H40	1.2339	0.3502	0.7700	0.062*	
C41	1.28442 (15)	0.19480 (12)	0.74656 (10)	0.0539 (4)	
H41	1.3594	0.2078	0.7024	0.065*	
C42	1.04227 (17)	0.46471 (12)	0.87027 (12)	0.0605 (4)	
H42A	1.1304	0.4780	0.8904	0.091*	
H42B	0.9730	0.5117	0.9012	0.091*	
H42C	1.0444	0.4784	0.8028	0.091*	
O1	0.72972 (14)	0.57721 (13)	0.95517 (10)	0.0940 (5)	
O2	0.64601 (10)	0.42965 (8)	0.79142 (7)	0.0555 (3)	
O3	1.02571 (10)	0.35905 (7)	0.56416 (6)	0.0474 (2)	
O4	1.20006 (12)	0.33432 (9)	0.45684 (8)	0.0693 (3)	
O5	1.32377 (13)	−0.07151 (11)	0.50820 (9)	0.0787 (4)	
O6	1.31154 (11)	−0.00128 (8)	0.73480 (7)	0.0594 (3)	
O7	0.95511 (10)	0.13252 (8)	0.94262 (7)	0.0516 (3)	
O8	0.77875 (13)	0.18204 (10)	1.04032 (9)	0.0789 (4)	

### Atomic displacement parameters (Å^2^)

**Table:**

	*U*^11^	*U*^22^	*U*^33^	*U*^12^	*U*^13^	*U*^23^
C1	0.0502 (8)	0.0476 (8)	0.0527 (8)	0.0014 (6)	0.0055 (6)	−0.0058 (6)
C2	0.0466 (8)	0.0448 (7)	0.0451 (7)	0.0007 (6)	0.0036 (6)	−0.0037 (6)
C3	0.0535 (9)	0.0591 (9)	0.0517 (8)	0.0054 (7)	−0.0029 (7)	−0.0043 (7)
C4	0.0750 (11)	0.0830 (12)	0.0516 (9)	0.0060 (9)	−0.0045 (8)	−0.0195 (8)
C5	0.0473 (8)	0.0498 (8)	0.0459 (7)	0.0000 (6)	0.0043 (6)	−0.0048 (6)
C6	0.0475 (8)	0.0588 (8)	0.0501 (8)	−0.0086 (7)	−0.0001 (6)	0.0018 (6)
C7	0.0688 (10)	0.0556 (9)	0.0614 (9)	−0.0117 (8)	−0.0117 (8)	0.0001 (7)
C8	0.0943 (14)	0.0721 (11)	0.0664 (11)	−0.0254 (10)	−0.0215 (10)	−0.0011 (9)
C9	0.0848 (14)	0.1025 (16)	0.0728 (12)	−0.0271 (12)	−0.0317 (11)	0.0141 (11)
C10	0.0601 (11)	0.1067 (16)	0.0838 (13)	0.0014 (10)	−0.0202 (10)	0.0108 (12)
C11	0.0510 (9)	0.0832 (12)	0.0686 (10)	0.0031 (8)	−0.0048 (8)	−0.0026 (9)
C12	0.0500 (8)	0.0476 (7)	0.0399 (7)	0.0048 (6)	−0.0011 (6)	−0.0051 (6)
C13	0.0512 (8)	0.0406 (7)	0.0422 (7)	0.0022 (6)	−0.0011 (6)	−0.0034 (5)
C14	0.0437 (7)	0.0458 (7)	0.0353 (6)	−0.0009 (5)	−0.0051 (5)	−0.0047 (5)
C15	0.0453 (8)	0.0547 (8)	0.0476 (7)	0.0017 (6)	−0.0002 (6)	−0.0146 (6)
C16	0.0503 (8)	0.0521 (8)	0.0507 (8)	0.0068 (6)	−0.0059 (6)	−0.0172 (6)
C17	0.0548 (8)	0.0447 (7)	0.0435 (7)	0.0034 (6)	−0.0144 (6)	−0.0114 (6)
C18	0.0499 (8)	0.0427 (7)	0.0378 (6)	0.0012 (6)	−0.0096 (6)	−0.0069 (5)
C19	0.0637 (9)	0.0408 (7)	0.0470 (7)	−0.0049 (6)	−0.0038 (6)	−0.0020 (6)
C20	0.0585 (9)	0.0526 (8)	0.0445 (7)	−0.0051 (7)	0.0059 (6)	−0.0012 (6)
C21	0.0819 (11)	0.0447 (8)	0.0599 (9)	0.0044 (7)	−0.0084 (8)	−0.0116 (7)
C22	0.0587 (9)	0.0510 (8)	0.0519 (8)	0.0034 (7)	0.0086 (7)	−0.0032 (6)
C23	0.0497 (8)	0.0493 (8)	0.0472 (7)	0.0031 (6)	0.0048 (6)	−0.0064 (6)
C24	0.0510 (8)	0.0594 (9)	0.0479 (8)	0.0018 (7)	0.0037 (6)	−0.0053 (7)
C25	0.0733 (11)	0.0645 (10)	0.0535 (9)	−0.0042 (8)	−0.0001 (8)	−0.0151 (7)
C26	0.0494 (8)	0.0457 (7)	0.0487 (8)	−0.0015 (6)	0.0044 (6)	−0.0050 (6)
C27	0.0550 (9)	0.0509 (8)	0.0526 (8)	−0.0075 (7)	−0.0026 (7)	0.0028 (6)
C28	0.0751 (11)	0.0753 (11)	0.0539 (9)	−0.0009 (9)	−0.0068 (8)	−0.0065 (8)
C29	0.1088 (17)	0.0907 (14)	0.0555 (10)	−0.0165 (12)	−0.0136 (10)	−0.0068 (9)
C30	0.0939 (16)	0.1127 (17)	0.0658 (12)	−0.0307 (13)	−0.0284 (11)	0.0124 (11)
C31	0.0666 (12)	0.1114 (17)	0.0821 (13)	−0.0081 (11)	−0.0208 (10)	0.0113 (12)
C32	0.0590 (10)	0.0730 (11)	0.0685 (10)	−0.0027 (8)	−0.0073 (8)	0.0041 (8)
C33	0.0518 (8)	0.0474 (7)	0.0426 (7)	0.0075 (6)	−0.0010 (6)	−0.0086 (6)
C34	0.0550 (8)	0.0432 (7)	0.0439 (7)	0.0044 (6)	−0.0006 (6)	−0.0037 (6)
C35	0.0454 (7)	0.0492 (7)	0.0348 (6)	0.0043 (6)	−0.0035 (5)	−0.0045 (5)
C36	0.0536 (9)	0.0573 (9)	0.0480 (8)	0.0108 (7)	0.0037 (7)	−0.0078 (6)
C37	0.0534 (8)	0.0515 (8)	0.0526 (8)	0.0113 (7)	−0.0036 (7)	−0.0145 (6)
C38	0.0500 (8)	0.0467 (7)	0.0441 (7)	0.0063 (6)	−0.0118 (6)	−0.0104 (6)
C39	0.0477 (8)	0.0450 (7)	0.0385 (7)	0.0036 (6)	−0.0086 (6)	−0.0076 (5)
C40	0.0570 (9)	0.0459 (8)	0.0511 (8)	−0.0029 (6)	−0.0004 (7)	−0.0054 (6)
C41	0.0533 (8)	0.0579 (9)	0.0486 (8)	−0.0013 (7)	0.0061 (6)	−0.0073 (6)
C42	0.0666 (10)	0.0456 (8)	0.0698 (10)	0.0040 (7)	−0.0065 (8)	−0.0132 (7)
O1	0.0745 (9)	0.1308 (12)	0.0787 (9)	0.0390 (8)	−0.0274 (7)	−0.0288 (8)
O2	0.0588 (6)	0.0479 (6)	0.0549 (6)	0.0049 (5)	0.0153 (5)	−0.0027 (4)
O3	0.0486 (5)	0.0452 (5)	0.0469 (5)	0.0001 (4)	0.0050 (4)	−0.0069 (4)
O4	0.0645 (7)	0.0653 (7)	0.0735 (7)	−0.0037 (6)	0.0229 (6)	−0.0136 (6)
O5	0.0785 (8)	0.0898 (9)	0.0687 (8)	0.0230 (7)	−0.0204 (6)	−0.0139 (6)
O6	0.0631 (7)	0.0521 (6)	0.0587 (6)	0.0062 (5)	0.0151 (5)	−0.0088 (5)
O7	0.0554 (6)	0.0472 (5)	0.0487 (5)	0.0064 (4)	0.0075 (4)	−0.0035 (4)
O8	0.0771 (8)	0.0693 (8)	0.0805 (8)	0.0095 (6)	0.0320 (7)	−0.0023 (6)

### Geometric parameters (Å, º)

**Table:**

C1—O2	1.4387 (17)	C22—O6	1.4281 (17)
C1—C2	1.4956 (18)	C22—C23	1.4992 (19)
C1—H1A	0.9700	C22—H22A	0.9700
C1—H1B	0.9700	C22—H22B	0.9700
C2—C5	1.3396 (19)	C23—C26	1.336 (2)
C2—C3	1.486 (2)	C23—C24	1.484 (2)
C3—O1	1.2085 (18)	C24—O5	1.2155 (18)
C3—C4	1.493 (2)	C24—C25	1.500 (2)
C4—H4A	0.9600	C25—H25A	0.9600
C4—H4B	0.9600	C25—H25B	0.9600
C4—H4C	0.9600	C25—H25C	0.9600
C5—C6	1.463 (2)	C26—C27	1.465 (2)
C5—H5	0.9300	C26—H26	0.9300
C6—C7	1.392 (2)	C27—C28	1.390 (2)
C6—C11	1.397 (2)	C27—C32	1.392 (2)
C7—C8	1.381 (2)	C28—C29	1.380 (2)
C7—H7	0.9300	C28—H28	0.9300
C8—C9	1.366 (3)	C29—C30	1.367 (3)
C8—H8	0.9300	C29—H29	0.9300
C9—C10	1.372 (3)	C30—C31	1.370 (3)
C9—H9	0.9300	C30—H30	0.9300
C10—C11	1.380 (3)	C31—C32	1.378 (3)
C10—H10	0.9300	C31—H31	0.9300
C11—H11	0.9300	C32—H32	0.9300
C12—O2	1.3586 (15)	C33—O6	1.3659 (16)
C12—C13	1.3808 (19)	C33—C34	1.381 (2)
C12—C20	1.397 (2)	C33—C41	1.390 (2)
C13—C14	1.3836 (18)	C34—C35	1.3744 (18)
C13—H13	0.9300	C34—H34	0.9300
C14—O3	1.3733 (16)	C35—O7	1.3739 (16)
C14—C18	1.3870 (19)	C35—C39	1.3908 (19)
C15—O4	1.2084 (18)	C36—O8	1.2070 (18)
C15—O3	1.3747 (16)	C36—O7	1.3748 (16)
C15—C16	1.430 (2)	C36—C37	1.432 (2)
C16—C17	1.346 (2)	C37—C38	1.340 (2)
C16—H16	0.9300	C37—H37	0.9300
C17—C18	1.4455 (18)	C38—C39	1.4479 (18)
C17—C21	1.493 (2)	C38—C42	1.491 (2)
C18—C19	1.398 (2)	C39—C40	1.386 (2)
C19—C20	1.3648 (19)	C40—C41	1.3797 (19)
C19—H19	0.9300	C40—H40	0.9300
C20—H20	0.9300	C41—H41	0.9300
C21—H21A	0.9600	C42—H42A	0.9600
C21—H21B	0.9600	C42—H42B	0.9600
C21—H21C	0.9600	C42—H42C	0.9600
			
O2—C1—C2	108.32 (11)	C23—C22—H22A	110.2
O2—C1—H1A	110.0	O6—C22—H22B	110.2
C2—C1—H1A	110.0	C23—C22—H22B	110.2
O2—C1—H1B	110.0	H22A—C22—H22B	108.5
C2—C1—H1B	110.0	C26—C23—C24	121.63 (13)
H1A—C1—H1B	108.4	C26—C23—C22	123.12 (13)
C5—C2—C3	120.75 (12)	C24—C23—C22	115.14 (13)
C5—C2—C1	124.21 (13)	O5—C24—C23	120.00 (14)
C3—C2—C1	115.04 (12)	O5—C24—C25	119.89 (14)
O1—C3—C2	119.53 (14)	C23—C24—C25	120.09 (13)
O1—C3—C4	119.44 (14)	C24—C25—H25A	109.5
C2—C3—C4	121.03 (13)	C24—C25—H25B	109.5
C3—C4—H4A	109.5	H25A—C25—H25B	109.5
C3—C4—H4B	109.5	C24—C25—H25C	109.5
H4A—C4—H4B	109.5	H25A—C25—H25C	109.5
C3—C4—H4C	109.5	H25B—C25—H25C	109.5
H4A—C4—H4C	109.5	C23—C26—C27	129.65 (13)
H4B—C4—H4C	109.5	C23—C26—H26	115.2
C2—C5—C6	130.26 (13)	C27—C26—H26	115.2
C2—C5—H5	114.9	C28—C27—C32	117.74 (15)
C6—C5—H5	114.9	C28—C27—C26	124.75 (14)
C7—C6—C11	117.75 (15)	C32—C27—C26	117.51 (14)
C7—C6—C5	124.07 (14)	C29—C28—C27	120.72 (18)
C11—C6—C5	118.12 (14)	C29—C28—H28	119.6
C8—C7—C6	120.84 (17)	C27—C28—H28	119.6
C8—C7—H7	119.6	C30—C29—C28	120.48 (19)
C6—C7—H7	119.6	C30—C29—H29	119.8
C9—C8—C7	120.53 (19)	C28—C29—H29	119.8
C9—C8—H8	119.7	C29—C30—C31	119.77 (19)
C7—C8—H8	119.7	C29—C30—H30	120.1
C8—C9—C10	119.74 (18)	C31—C30—H30	120.1
C8—C9—H9	120.1	C30—C31—C32	120.3 (2)
C10—C9—H9	120.1	C30—C31—H31	119.8
C9—C10—C11	120.50 (19)	C32—C31—H31	119.8
C9—C10—H10	119.7	C31—C32—C27	120.87 (18)
C11—C10—H10	119.7	C31—C32—H32	119.6
C10—C11—C6	120.63 (18)	C27—C32—H32	119.6
C10—C11—H11	119.7	O6—C33—C34	114.83 (12)
C6—C11—H11	119.7	O6—C33—C41	125.12 (13)
O2—C12—C13	124.43 (12)	C34—C33—C41	120.05 (12)
O2—C12—C20	115.23 (12)	C35—C34—C33	118.86 (13)
C13—C12—C20	120.34 (12)	C35—C34—H34	120.6
C12—C13—C14	117.79 (12)	C33—C34—H34	120.6
C12—C13—H13	121.1	O7—C35—C34	115.89 (12)
C14—C13—H13	121.1	O7—C35—C39	121.17 (12)
O3—C14—C13	115.28 (11)	C34—C35—C39	122.94 (13)
O3—C14—C18	121.25 (11)	O8—C36—O7	115.77 (14)
C13—C14—C18	123.47 (12)	O8—C36—C37	126.60 (13)
O4—C15—O3	115.98 (13)	O7—C36—C37	117.63 (13)
O4—C15—C16	126.84 (13)	C38—C37—C36	122.93 (13)
O3—C15—C16	117.17 (12)	C38—C37—H37	118.5
C17—C16—C15	122.92 (13)	C36—C37—H37	118.5
C17—C16—H16	118.5	C37—C38—C39	118.30 (13)
C15—C16—H16	118.5	C37—C38—C42	122.24 (13)
C16—C17—C18	118.49 (13)	C39—C38—C42	119.46 (13)
C16—C17—C21	121.99 (13)	C40—C39—C35	116.64 (12)
C18—C17—C21	119.52 (13)	C40—C39—C38	124.60 (13)
C14—C18—C19	116.91 (12)	C35—C39—C38	118.75 (13)
C14—C18—C17	118.52 (13)	C41—C40—C39	121.97 (14)
C19—C18—C17	124.56 (13)	C41—C40—H40	119.0
C20—C19—C18	121.14 (13)	C39—C40—H40	119.0
C20—C19—H19	119.4	C40—C41—C33	119.50 (13)
C18—C19—H19	119.4	C40—C41—H41	120.3
C19—C20—C12	120.32 (13)	C33—C41—H41	120.3
C19—C20—H20	119.8	C38—C42—H42A	109.5
C12—C20—H20	119.8	C38—C42—H42B	109.5
C17—C21—H21A	109.5	H42A—C42—H42B	109.5
C17—C21—H21B	109.5	C38—C42—H42C	109.5
H21A—C21—H21B	109.5	H42A—C42—H42C	109.5
C17—C21—H21C	109.5	H42B—C42—H42C	109.5
H21A—C21—H21C	109.5	C12—O2—C1	118.01 (11)
H21B—C21—H21C	109.5	C14—O3—C15	121.63 (11)
O6—C22—C23	107.67 (12)	C33—O6—C22	117.14 (11)
O6—C22—H22A	110.2	C35—O7—C36	121.20 (11)
			
O2—C1—C2—C5	−89.47 (16)	C22—C23—C26—C27	2.4 (2)
O2—C1—C2—C3	90.67 (15)	C23—C26—C27—C28	26.9 (3)
C5—C2—C3—O1	171.46 (16)	C23—C26—C27—C32	−153.46 (16)
C1—C2—C3—O1	−8.7 (2)	C32—C27—C28—C29	3.4 (3)
C5—C2—C3—C4	−7.6 (2)	C26—C27—C28—C29	−177.02 (16)
C1—C2—C3—C4	172.22 (14)	C27—C28—C29—C30	−1.5 (3)
C3—C2—C5—C6	−174.56 (14)	C28—C29—C30—C31	−1.3 (3)
C1—C2—C5—C6	5.6 (2)	C29—C30—C31—C32	2.0 (3)
C2—C5—C6—C7	26.7 (2)	C30—C31—C32—C27	0.0 (3)
C2—C5—C6—C11	−156.43 (16)	C28—C27—C32—C31	−2.6 (3)
C11—C6—C7—C8	0.2 (2)	C26—C27—C32—C31	177.72 (16)
C5—C6—C7—C8	177.05 (15)	O6—C33—C34—C35	−176.91 (12)
C6—C7—C8—C9	−0.3 (3)	C41—C33—C34—C35	2.6 (2)
C7—C8—C9—C10	0.9 (3)	C33—C34—C35—O7	178.55 (12)
C8—C9—C10—C11	−1.3 (3)	C33—C34—C35—C39	−1.6 (2)
C9—C10—C11—C6	1.2 (3)	O8—C36—C37—C38	178.55 (16)
C7—C6—C11—C10	−0.6 (2)	O7—C36—C37—C38	−1.0 (2)
C5—C6—C11—C10	−177.68 (16)	C36—C37—C38—C39	1.2 (2)
O2—C12—C13—C14	177.87 (12)	C36—C37—C38—C42	−178.88 (14)
C20—C12—C13—C14	−1.7 (2)	O7—C35—C39—C40	179.56 (12)
C12—C13—C14—O3	−178.97 (11)	C34—C35—C39—C40	−0.3 (2)
C12—C13—C14—C18	1.4 (2)	O7—C35—C39—C38	−1.25 (19)
O4—C15—C16—C17	−177.92 (15)	C34—C35—C39—C38	178.93 (12)
O3—C15—C16—C17	1.6 (2)	C37—C38—C39—C40	179.06 (13)
C15—C16—C17—C18	−0.2 (2)	C42—C38—C39—C40	−0.9 (2)
C15—C16—C17—C21	179.40 (13)	C37—C38—C39—C35	−0.06 (19)
O3—C14—C18—C19	−179.87 (11)	C42—C38—C39—C35	−179.99 (12)
C13—C14—C18—C19	−0.22 (19)	C35—C39—C40—C41	1.2 (2)
O3—C14—C18—C17	0.99 (18)	C38—C39—C40—C41	−177.95 (13)
C13—C14—C18—C17	−179.35 (12)	C39—C40—C41—C33	−0.2 (2)
C16—C17—C18—C14	−1.07 (19)	O6—C33—C41—C40	177.74 (13)
C21—C17—C18—C14	179.32 (12)	C34—C33—C41—C40	−1.7 (2)
C16—C17—C18—C19	179.86 (13)	C13—C12—O2—C1	−3.1 (2)
C21—C17—C18—C19	0.3 (2)	C20—C12—O2—C1	176.50 (12)
C14—C18—C19—C20	−0.6 (2)	C2—C1—O2—C12	−173.35 (12)
C17—C18—C19—C20	178.49 (13)	C13—C14—O3—C15	−179.27 (11)
C18—C19—C20—C12	0.2 (2)	C18—C14—O3—C15	0.42 (18)
O2—C12—C20—C19	−178.64 (13)	O4—C15—O3—C14	177.88 (12)
C13—C12—C20—C19	1.0 (2)	C16—C15—O3—C14	−1.65 (18)
O6—C22—C23—C26	−88.27 (17)	C34—C33—O6—C22	176.75 (12)
O6—C22—C23—C24	95.48 (15)	C41—C33—O6—C22	−2.7 (2)
C26—C23—C24—O5	−177.42 (15)	C23—C22—O6—C33	−178.10 (12)
C22—C23—C24—O5	−1.1 (2)	C34—C35—O7—C36	−178.73 (12)
C26—C23—C24—C25	1.2 (2)	C39—C35—O7—C36	1.44 (19)
C22—C23—C24—C25	177.53 (13)	O8—C36—O7—C35	−179.95 (13)
C24—C23—C26—C27	178.41 (14)	C37—C36—O7—C35	−0.3 (2)

### Hydrogen-bond geometry (Å, º)

*Cg*1 is centroid of the C6–C11 ring.

**Table:**

*D*—H···*A*	*D*—H	H···*A*	*D*···*A*	*D*—H···*A*
C5—H5···O8^i^	0.93	2.59	3.4778 (19)	159
C9—H9···O3^ii^	0.93	2.52	3.381 (2)	155
C30—H30···O7^iii^	0.93	2.59	3.441 (2)	153
C42—H42*B*···O1	0.96	2.53	3.427 (2)	155
C25—H25*C*···*Cg*1^iv^	0.96	2.64	3.5238 (18)	152

Symmetry codes: (i) −*x*+1, −*y*+1, −*z*+2; (ii) −*x*+1, −*y*+1, −*z*+1; (iii) −*x*+3, −*y*, −*z*+2; (iv) *x*+1, *y*−1, *z*.
