# Supplementary figures and images for: Crystal structure of 7-[(2E)-2-benzyl­idene-3-oxobut­oxy]-4-methyl-2H-chromen-2-one
Source: Acta Crystallogr E Crystallogr Commun. 2015 Mar 4;71(Pt 4):o222–3. doi: 10.1107/S2056989015003084 (PMC4438806; doi:10.1107/S2056989015003084)

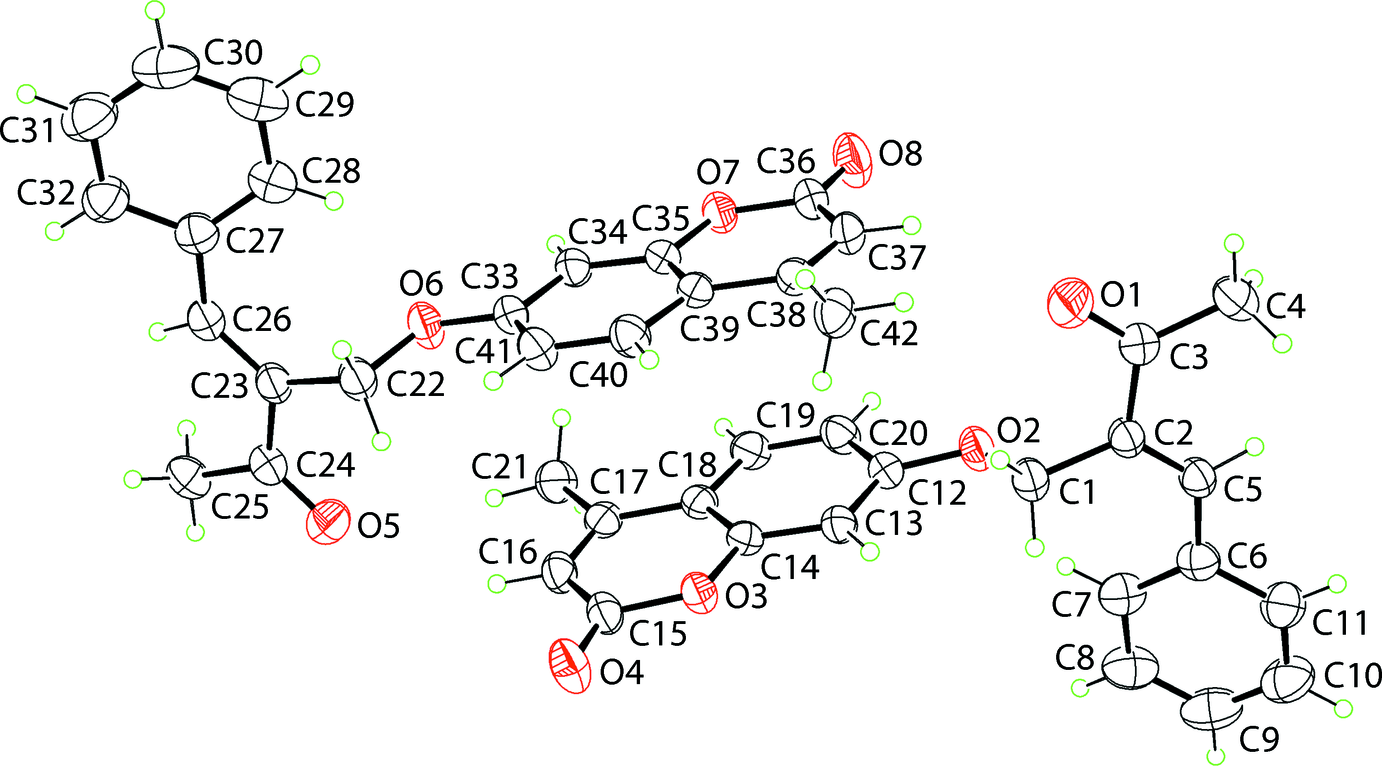

Supplement: Supplementary file 4 [file e-71-0o222-fig1.tif]

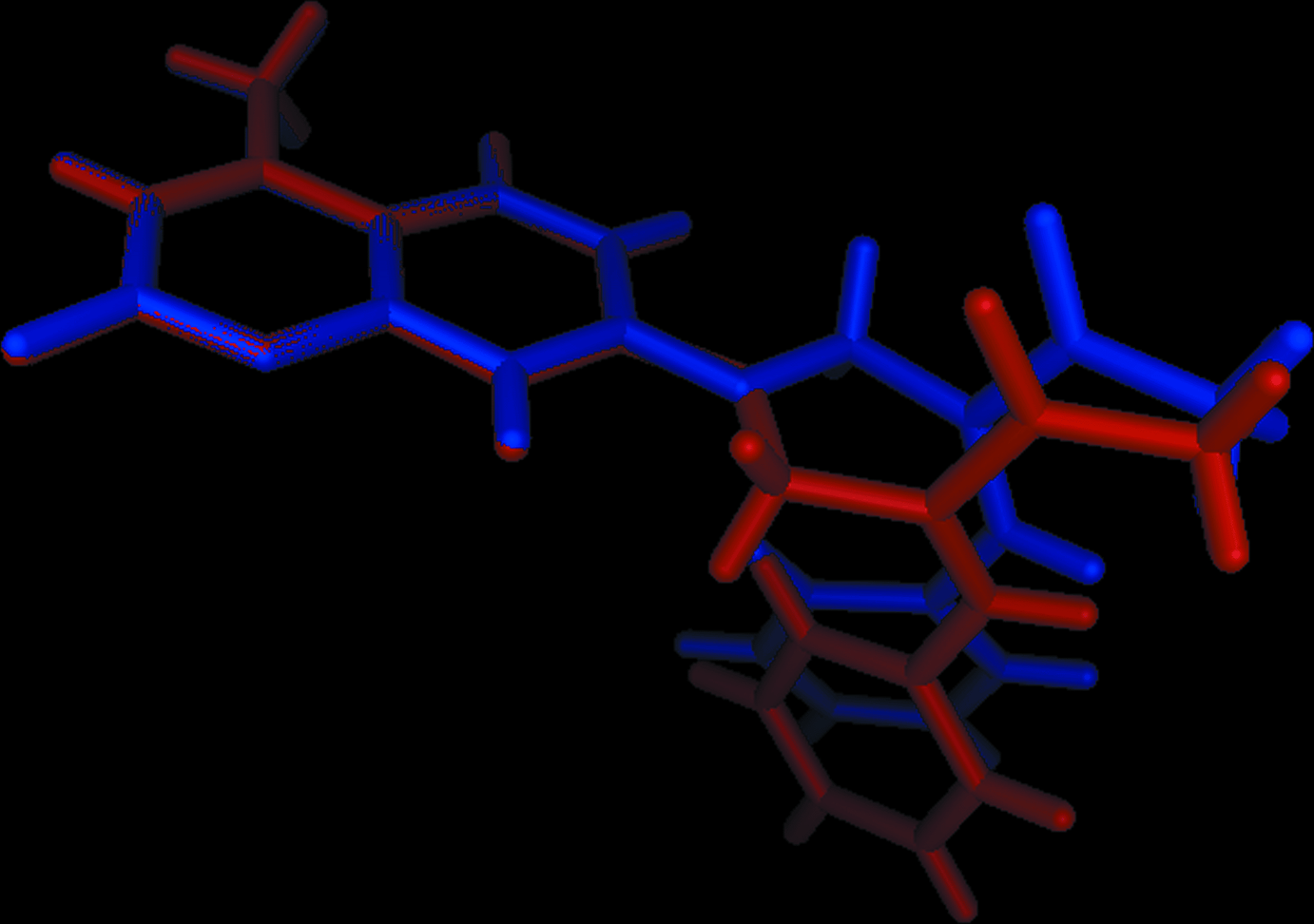

Supplement: Supplementary file 5 [file e-71-0o222-fig2.tif]

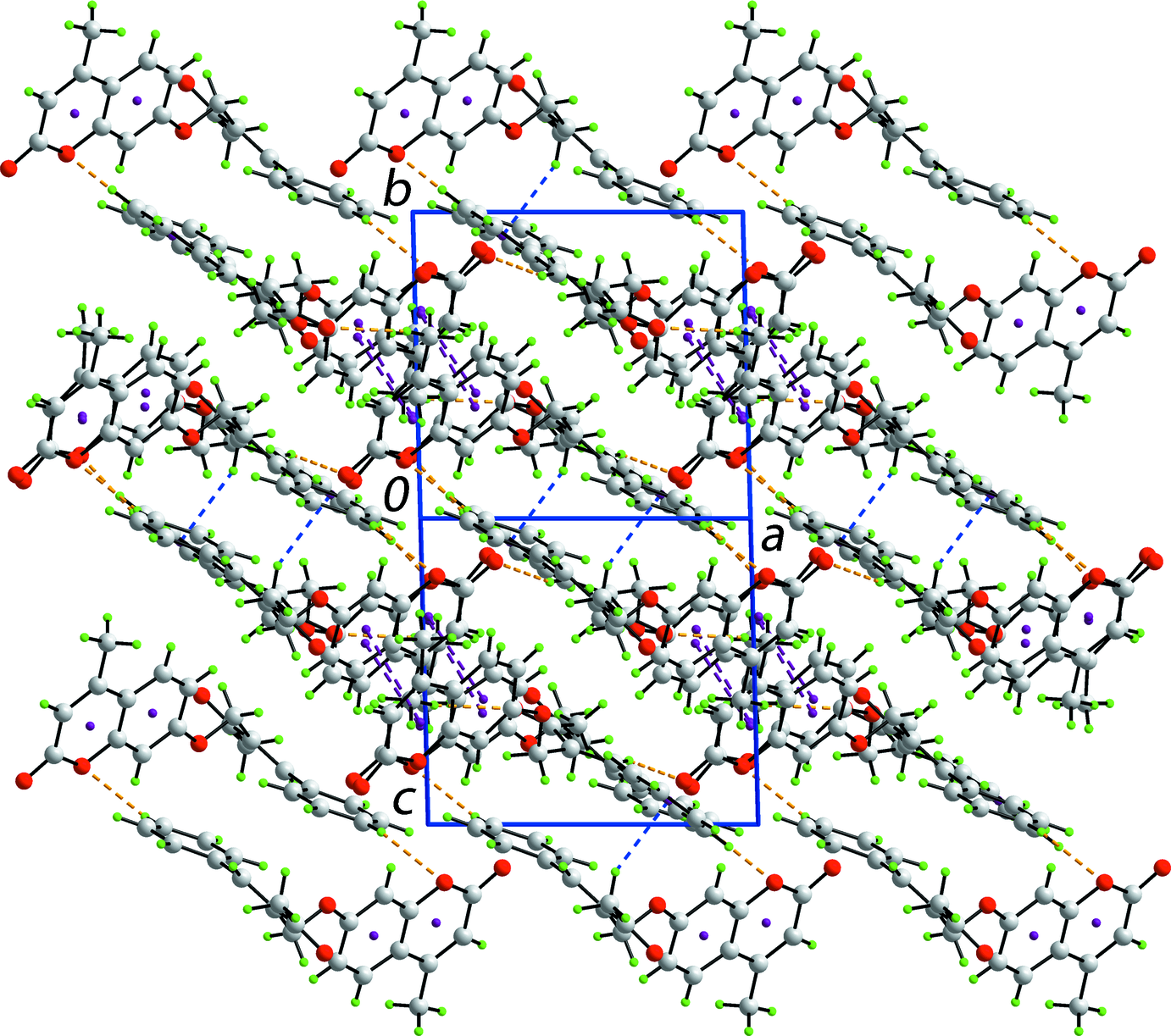

Supplement: Supplementary file 6 [file e-71-0o222-fig3.tif]
